# Inhibition of colon cancer K-Ras^G13D^ mutation reduces cancer cell proliferation but promotes stemness and inflammation *via* RAS/ERK pathway

**DOI:** 10.3389/fphar.2022.996053

**Published:** 2022-10-28

**Authors:** Yan Qi, Hong Zou, XiaoHui Zhao, Joanna Kapeleris, Michael Monteiro, Feng Li, Zhi Ping Xu, Yizhen Deng, Yanheng Wu, Ying Tang, Wenyi Gu

**Affiliations:** ^1^ Department of Pathology, Central People’s Hospital of Zhanjiang and Zhanjiang Central Hospital, Guangdong Medical University, Zhanjiang, China; ^2^ Australian Institute for Bioengineering and Nanotechnology (AIBN), University of Queensland (UQ), Brisbane, QLD, Australia; ^3^ Department of Pathology, The Second Affiliated Hospital of Zhejiang University School of Medicine, Hangzhou, China; ^4^ Department of Pathology, Beijing Chaoyang Hospital, Capital Medical University, Beijing, China; ^5^ Gillion Biotherapeutics Ltd., Guangzhou Huangpu Industrial Zoon, Guangzhou, China; ^6^ Science and Technology Innovation Center, Guangzhou University of Chinese Medicine, Guangzhou, China

**Keywords:** K-Ras^G13D^ mutation, colon cancer, cancer stem cells, tumor spheroid, ERK pathway, PI3K/Akt pathway, inflammation

## Abstract

K-Ras is a well-studied oncogene, and its mutation is frequently found in epithelial cancers like pancreas, lung, and colorectal cancers. Cancer cells harboring K-Ras mutations are difficult to treat due to the drug resistance and metastasis properties. Cancer stem cells (CSCs) are believed the major cause of chemotherapeutic resistance and responsible for tumor recurrence and metastasis. But how K-Ras mutation affects CSCs and inflammation is not clear. Here, we compared two colon cancer cell lines, HCT-116 and HT-29, with the former being K-Ras^G13D^ mutant and the latter being wildtype. We found that HCT-116 cells treated with a K-Ras mutation inhibitor S7333 formed significantly more tumor spheroids than the untreated control, while the wild type of HT-29 cells remained unchanged. However, the size of tumor spheroids was smaller than the untreated controls, indicating their proliferation was suppressed after S7333 treatment. Consistent with this, the expressions of stem genes Lgr5 and CD133 significantly increased and the expression of self-renewal gene TGF-β1 also increased. The flow cytometry analysis indicated that the expression of stem surface marker CD133 increased in the treated HCT-116 cells. To understand the pathway through which the G13D mutation induced the effects, we studied both RAS/ERK and PI3K/Akt pathways using specific inhibitors SCH772984 and BEZ235. The results indicated that RAS/ERK rather than PI3K/Akt pathway was involved. As CSCs play the initial role in cancer development and the inflammation is a vital step during tumor initiation, we analyzed the correlation between increased stemness and inflammation. We found a close correlation of increased Lgr5 and CD133 with proinflammatory factors like IL-17, IL-22, and IL-23. Together, our findings suggest that K-Ras^G13D^ mutation promotes cancer cell growth but decreases cancer stemness and inflammation thus tumorigenesis and metastasis potential in colon cancer. Inhibition of this mutation reverses the process. Therefore, care needs be taken when employing targeted therapies to K-Ras^G13D^ mutations in clinics.

## Introduction

Gene mutation is frequently observed in tumor development and is one of the most common reasons for tumor aggressiveness and poor prognosis (Chen et al., 2021), which is also known as tumor mutation burden (TMB). High TMB tumors are usually correlated with the deficiency of DNA repair, microsatellite instability, and a high possibility of drug resistance though they may attract more immune cell infiltration (Chen et al., 2021; Xiao et al., 2021). K-Ras gene is one of the most popular and well-defined oncogene that mutates in a broad range of solid tumors (Der et al., 1982), including lung adenocarcinoma (∼30%) (Siegfried et al., 1997), pancreatic adenocarcinoma (∼70%–90%) (Visani et al., 2013), stomach cancer (∼10%) (Lee et al., 2003), and colorectal cancer (∼30%–50%) (Andreyev et al., 1998; Downward, 2003). K-Ras mutations are generally associated with poor overall survival and resistance to therapies (Downward, 2003) and these mutations often occur at codons 12, 13, and 61, with codon 12 being the most frequently mutated (Addeo et al., 2021). The mutations at codons 12 and 13 can lead to wild-type glycine (G) being replaced by cysteine (C), valine (V), aspartic acid (D), arginine (R), alanine (A), or serine (S) (Cox et al., 2014). These mutations are usually companied with alternations of signaling pathways and thus change cancer cells in proliferation, drug resistance, and tumor recurrence. Therefore, in clinical settings, detection of these mutations becomes an important tool for prognosis and guides for targeted cancer therapies.

Colon cancer is a top lethal cancer for men and women worldwide (Selvam et al., 2019; Lichtenstern et al., 2020) and K-Ras mutation is one of the most frequently activated oncogenes observed in around 40% of colorectal carcinomas (Morkel et al., 2015). In clinical settings, once K-Ras gene mutations are detected, the inhibitor treatment of the gene mutation (targeted therapy) will be applied together with first line chemotherapeutics (Reck et al., 2021; Serna-Blasco et al., 2021). However, even with the targeted therapy, concerns still exist regarding the effectiveness of such therapies *in vivo* given the possibilities of existing intratumor heterogeneity or *de novo* mutation leading to treatment resistance (Cannataro et al., 2018) and the 5-year overall survival rate of colon cancer is still low around 10% (Davies and Goldberg, 2011). Consequently, K-Ras-specific drugs have rarely been approved in clinics (Christensen et al., 2020), suggesting whether the use of mutation inhibitors will benefit the long-term outcome of treatment and the deep mechanism of K-Ras mutations needs further investigations.

Cancer stem cells (CSCs) are a small population of cancer cells with multi-potency and self-renewing abilities. They are the “seeds” of tumors, responsible for the initiation, growth, and development of tumors *in vivo* (Gu et al., 2011; Gu et al., 2015). From this concept, the gene mutation may firstly occur in CSCs and then heritage to the daughter cells. Therefore, understanding the effect of oncogene mutation such as K-Ras in colon CSCs is very important for the future development of more effective approaches for targeted therapies. Meanwhile, understanding the relationship between oncogene mutation and cancer stemness, especially how stemness will be changed after the inhibition of the effect of a gene mutation, is an interesting and open question to answer. For CSCs, there is no unique biomarker to identify them, for instance, in colon cancer, the most popular biomarkers are some surface adhesion molecules like CD24, CD44, and CD133 (Chen et al., 2015; Lee et al., 2015). In addition, some typical stem genes like CD133 and Lgr5, and self-renewing genes like TGF-β are also used to characterize colon CSCs (Gu et al., 2011; Chen et al., 2015).

It has been reported that K-Ras mutation can activate different pathways and thus affect cancer cell proliferation and development. For example, about 40% of colorectal cancers have mutations in K-Ras accompanied by the downstream activation of mitogen-activated protein kinase (MAPK) signalling, which promotes tumour invasion and progression since tumour cells with high MAPK activity resided specifically at the leading tumour edge, ceased to proliferate, underwent epithelial-mesenchymal transition (EMT), and expressed markers related to colon CSCs (Blaj et al., 2017). These results imply that differential MAPK signalling balances EMT, cancer stem cell potential, and tumour growth in colorectal cancer (Blaj et al., 2017).

Compared with other K-Ras mutations, G12 mutation is the most frequent and well-studied, which was also shown to relate to drug resistance in non-small-lung carcinoma (Adachi et al., 2020). Recently, an inhibitor, sotorasib, has been approved by the Food and Drug Administration as a fast track designation for the treatment of metastatic non-small-cell lung carcinoma with the G12C KRAS mutation (FDA website: published on Fri 28 May 2021). A study analysed tissues from colorectal (CRC) patients (*n* = 49) to determine whether K-Ras mutations contributed to CSC activation during colorectal tumorigenesis. K-Ras wild-type DLD-1-K-Ras-WT and K-Ras mutated DLD-1-K-Ras-MT cells were cultured and evaluated for their ability to differentiate, form spheroids *in vitro*, and form tumours *in vivo*. Interaction between APC (Adenomatous polyposis *coli*) and K-Ras mutations in colorectal tumorigenesis was evaluated using APC (Min/+)/K-Ras (LA2) mice and DLD-1-K-Ras-WT and DLD-1-K-Ras-MT cell xenografts. The results showed that the sphere-forming capability of DLD-1-K-Ras-MT cells was significantly higher than that of DLD-1-K-Ras-WT cells (DLD-1-K-Ras-MT mean = 86.661 pixel, DLD-1-K-Ras-WT mean = 42.367 pixels, *p* = 0.003). Moreover, both the size and weight of tumours from DLD-1-K-Ras-MT xenografts were markedly increased compared with tumours from DLD-1-K-Ras-WT cells. Expression of the CSC markers CD44, CD133, and CD166 was induced in intestinal tumours from APC (Min/+)/K-Ras (LA2) mice, but not K-Ras (LA2) mice, indicating that K-Ras mutated cells are more tumorigenic and that APC mutation is required for CSC activation by oncogenic K-Ras mutation (Moon et al., 2014).

Unlike 12C, K-Ras G13 mutation is much less studied and whether K-Ras G13 point mutation is also related to cancer stemness, especially when the mutation is targeted, whether the signal pathway change will alter the stemness in colon cancer has not been reported before. In this research, we aimed to study the relation of K-Ras G13 mutation with cancer stemness. HCT-116 and HT-29 cells are two well-established colon cancer cell lines and reported to be K-Ras G13D mutated and wild type, respectively (Ahmed et al., 2013). We, therefore, employ an inhibitor of K-Ras G12C, which could also block G13 mutation at a higher dose or long-term treatment, to study if suppressing the mutation will affect the stemness of colon HCT-116 cells. We found that suppressing this mutation can reduce colon cancer cell proliferation but increase their stemness, including sphere formation and stem surface and gene marker expressions. We further prove that this effect is through the RAS/ERK pathway. To further confirm the increased stemness will affect tumor recurrence and metastasis, we analyzed their relations to inflammation, we found a good correlation with pro-inflammatory factors like IL-17, IL-22, and IL-23, which are reported as important factors for tumor initiation (Grivennikov et al., 2012; Zhao et al., 2021). These results indicate that when using targeted therapies for K-Ras G13 mutation, we may need to consider its effects on promoting cancer stemness and inflammation.

## Materials and methods

### Cell lines and tumor spheroid culture

The colon cancer cell lines HCT-116 (K-Ras mutated at G13D) and HT-29 (wild type) were purchased from America Tissue Collection Centre (ATCC) as we reported before (Zou et al., 2016) and were maintained in Dulbecco’s Modified Eagles Medium (DMEM, Invitrogen, Australia) supplemented with 10% fetal calf serum, 1% penicillin, and 1% streptomycin in 75 ml flasks at 37°C and with 5% CO_2_. Sphere culture medium and method were as previously reported (Gu et al., 2011). Briefly, HCT-116 or HT-29 cells were suspended in sphere cultural media (Dulbecco’s Modified Eagle’s Medium F-12, 0.4% BSA, 0.2% epidermal growth factor and 0.2% insulin, Sigma) at a cell concentration of 4000 cells/mL in upright T25 flasks. The sphere culture was maintained in a humidified incubator at 37°C under 5% CO_2_. And fed every 2 days with 20% of the original culture volume of sphere media until day 7–8. Spheroids were collected by centrifugation at 100× g for 5 min and resuspended in a sphere culture medium for counting (a cutoff size 50 µm) or trypsinized for 5 min to separate individual spherical cells by multiple pipetting and passing through 40 μm cell strainers. Isolated and purified spherical cells were further cultured in a sphere cultural medium for second generation culture or used for characterization and other assays. For counting or imaging the spheres, a small aliquot (eg, 50 μL) was transferred to a well of 96-well plate. After setting for 2 min, the spheroids in each well were counted and imaged. The total spheroid number was calculated by multiplying the count/well with the total volume (μL)/50 μL. The sphere size was calculated by total spherical cell numbers divided by spheroid numbers.

### Inhibitor treatment in cell culture

To evaluate the inhibitor effect on the number and size of tumor sphere formation, tumor cells (HCT-116 and HT-29) were cultured with or without S7333 inhibitor (4µM, K-Ras (G12C) inhibitor 6, Cat No: S7333, Jomar Life Research, Australia) for 72 h, SCH772984 inhibitor (25, 50, 100, 200 nM, R & D Systems, Inc., Minneapolis, MN, United States) for 48 h, and BEZ235 inhibitor (50, 100, 200, 300, 400 nM, Jomar Life Research, Australia) for 48 h in completed DMEM medium. After each treatment, cells were directly counted (trypan blue assay), used for MTT assay, and collected for real-time qRT-PCR tests and tumor spheroid culture as above. For combinational treatment, the cells were firstly treated with 4 µM S7333 for 72 h and then collected and set for further culture with the SCH772984 inhibitor (200 nM) or BEZ235 inhibitor (50 nM) for 48 h for gene testing or tumor spheroid culture for testing sphere formation and stemness surface markers.

### Flow cytometry analysis for colon cancer stemness surface markers

The spherical cells isolated from tumor spheroids of HCT-116 and HT-29 cell cultures without any treatment were firstly verified by their surface markers as we reported before (Wu et al., 2017). This was to ensure the colon CSCs have been enriched after the sphere culture. For the tumor spheroid culture after each treatment, 1 × 10^5^ spherical cells were used for staining with rabbit anti-human CD133 (prominin-1) antibody (Sigma-Aldrich); mouse anti-human CD44 conjugated with FITC (Invitrogen, Australia); and mouse anti-human CD24 antibody conjugated with RPE (Invitrogen, Australia). For CD133 staining, mouse anti-rabbit IgG-FITC (Sigma-Aldrich) was used as the secondary antibody. After 3 washes with 2% FCS/PBS, the cells were fixed in 2% paraformaldehyde (PFA)/PBS and analyzed by flow-cytometry (Accuri, BD) and CFlow Sampler software. Three biological repeats were performed.

### Real-time RT-PCR for stemness gene expression

Total RNA extraction from tumor cells was prepared as instructed by the manufacturer using TRIzol^®^ reagent (Invitrogen, Australia). Reverse transcription reactions were performed with oligo-dT primer using the High-Capacity cDNA RT Kit (Applied Biosystems). Real-time PCR was carried out with SYBR green master mixture (Promega) on a Rotor-Gene RG-3000 (Corbett Research, Australia) with the program pre-heating 95°C 5 min; then 40 cycles of 94°C 15 s; 60°C 15 s; and 72°C 20 s. The primers are: GAPDH, Forward: 5′-CTT​TTG​CGT​CGC​CAG-3′, Reverse: 5′-TTG​ATG​GCA​ACA​ATA​TCC​AC-3’; CD133, Forward 5′-CAC​CAA​GCA​CAG​AGG​GTC​AT-3′, Reverse 5′-CAC​TAC​CAA​GGA​CAA​GGC​GT-3’; TGF-β1, Forward 5′-CAA​CAA​TTC​CTG​GCG​ATA​CC-3′, Reverse 5′-GAA​CCC​GTT​GAT​GTC​CAC​TT-3’; Lgr5, Forward 5′- GAT​GTT​GCT​CAG​GGT​GGA​CT-3′, Reverse 5′- GGG​AGC​AGC​TGA​CTG​ATG​TT-3’; K-Ras, Forward 5′-TGT CAA​GCT​CAG​CAC​AAT​CTG, Reverse 5′-GGT​AGG​GAG​GCA​AGA​TGA​CA; Ki-67: Forward 5′-AAT​TCA​GAC​TCC​ATG​TGC​CTG​AG-3′, and Reverse 5′-CTT​GAC​ACA​CAC​ACA​TTG​TCC​TCA​GC-3′. The GAPDH primers were used as the internal control to normalize other gene expressions. The normalized expressions of treated samples were then used to calculate fold changes by dividing the expression of the control sample.

### MTT 3-(4,5-dimethylthiazol-2-yl)-2,5-diphenyltetrazolium bromide) assay

For cell survival assay, 1 × 10^4^ treated or untreated colon cancer cells in 100 μL of medium were seeded to each well in a 96-well plate and treated with various concentrations of BEZ235. After 24 h treatment, the MTT assay was used to measure cell viability as instructed by the manufacturer (Sigma-Aldrich). Briefly, after cells were incubated with 10 μL of MTT (5 mg/ml) for 4 h at 37°C under light-blocking conditions, the medium was then removed and 100 μL of DMSO was added into each well. The absorbance of each well was measured at 570/630 nm using the INFINITE M PLEX reader (Tecan). The 100% viable rates were cells with a complete DMEM medium.

### Western blotting analysis

Treated or untreated cells from the cultures were lysed in RIPA buffer (Cell Signal Technology) containing 2 μl/ml protease inhibitor cocktail (Sigma-Aldrich). Protein samples were separated by electrophoresis using pre-casted mini-PAGE (Bio-Rad) at 120 V for 1.5 h. The separated proteins were transferred onto the PVDF membrane at 100 V for 1 h. The membrane was blocked at room temperature with 5% bovine serum albumin in Tris-Buffered Saline and 0.5% Tween 20 (TBST) buffer for 1 h and washed three times with TBST with each wash being 5 min. The membranes then were incubated overnight with anti-human ERK1/ERK2 antibodies (Phospho-ERK1/ERK2 (Thr185, Tyr187) antibody (44-680G) and ERK1/ERK2 monoclonal antibody (ERK-7D8), ThermoFisher Scientific) at 1:1000 dilutions. After washing three times with TBST, the membrane was incubated for 2 h at room temperature with horseradish peroxidase conjugated goat anti-rabbit antibody (Cell Signal Technology) at a dilution of 1:2500. All the incubation was performed with a constant shaking. Then the membrane was rinsed 3 times with TBST, and merged in 2 mL Clarity ECL blotting substrate (Bio-Rad Laboratories, Inc., Hercules, CA) for 1 min. The blot image of antibody signals was acquired using Geldoc (Bio-Rad Laboratories, Inc., Hercules, CA). Densitometry analysis of protein bands was conducted by ImageJ software.

### Database analysis

The correlation of K-Ras expression with RAF, PI3K, AKT, MAPK, and IL-17 in the TCGA-COAD cohort was analyzed using Spearman’s correlation analysis. To analyze the K-Ras expression with stemness, the expression matrix was directly obtained through the GEO (http://www.ncbi.nlm.nih.gov/geo) Query package. After downloading and installing the Gene Set Enrichment Analysis Database (GSEA) software (http://software.broadinstitute.org/gsea/msigdb/index.jsp), the expression file, the description file, and the target cluster of the selected research need to be prepared and the parameters need to be set up to run GSEA. The enrichment analysis is performed based on the expression of K-Ras in the selected sample, and the result is all the enrichment of expressed genes in various metabolic pathways. It is generally believed that the absolute value of NES ≧ 1.0, p-val ≦ 0.05, and p. ajust ≦ 0.05 are meaningful gene sets. The K-Ras mRNA expression data were extracted from The Cancer Genome Atlas (TCGA) database (https://portal.gdc.cancer.gov/repository). The inclusion criteria are: 1) colon cancer; 2) complete RNA-seq data. A total of 480 colon cancer cases and 41 normal cases were included in the present study, and the workflow type was HTSeq-FPKM. To explore the correlation of expression of CD133 and IL-7, IL-17, IL-22, and IL-23 in the TCGA-COAD cohort was analyzed using Spearman’s correlation analysis. To explore the correlation of expression of LGR5 and IL-11, IL-17, IL-22, and IL-23 in the TCGA-COAD cohort was analyzed using Spearman’s correlation analysis.

### Data presentation and statistical analysis

Data collected from experimental and control groups were expressed as mean ± SD and these assays were repeated 2-3 times. The one-way analysis of variance and Turkey’s multiple comparisons or the unpaired Student’s t-test (GraphPad Prism 9.1 program) were used to analyze the differences between groups and discriminate the significant differences (two-tail, *p* < 0.05) between experimental and control groups.

## Results

### Inhibition of K-Ras^G13D^ mutation decreases HCT-116 cell proliferation but increases their sphere formation

**FIGURE 1:**
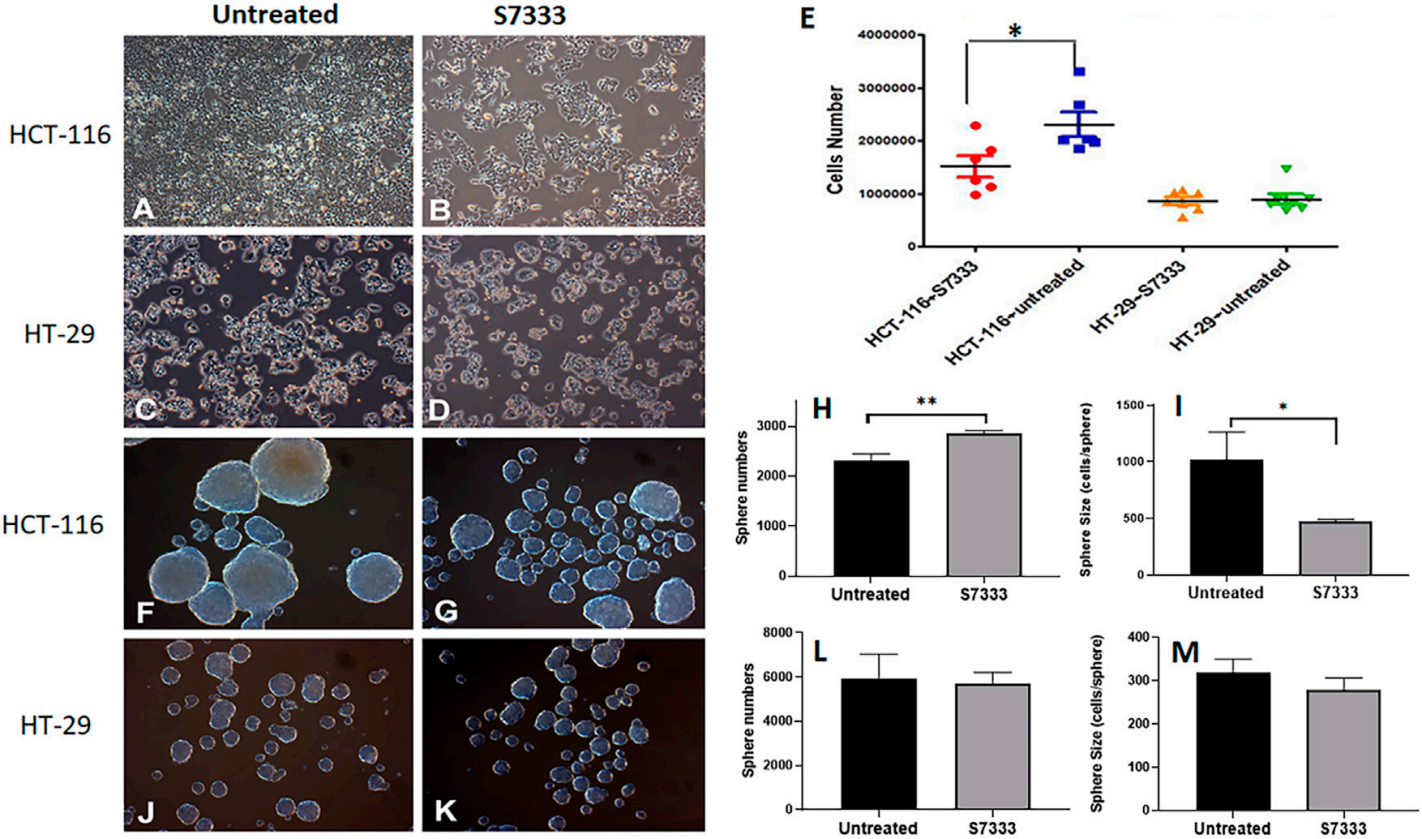
The comparison of cell growth and spheroid formation between HCT-116 and HT-29 cells after S7333 treatment. Cell morphology and density changes under light microscope of HCT-116 **(A,B)** and HT-29 cells **(C,D)** before and after treatment with S7333 for 72 h at 4 µM. **(E)**, the comparison of live cell numbers between HCT-116 and HT-29 cells before and after the treatment with S7333. **(F,G)** show the morphology and numbers of tumor spheroids from HCT-116 cells before **(F)** and after **(G)** treatment. The number of spheroids significantly increased **(H)** while the size of spheroids (average cell numbers/spheroid) significantly decreased **(I)** after S7333 treatment in HCT-116 cells. In HT-29 cells, no morphology **(J,K)**, number **(L)**, and size **(M)** changes were seen before and after S7333 treatment. *: *p* < 0.05; **: *p* < 0.01.

The K-Ras (G12C) inhibitor 6-S7333 (named S7333 afterward) was employed according to the manufacturers’ instruction, in which, the treatment lasted for 3 days (72 h) would inhibit the activity of G12/G13 mutations. Therefore, both cell lines of HCT-116 (K-Ras G13D mutated, K-Ras^G13D^) and HT-29 (wide type, K-Ras^WT^) were treated with S7333 for 72 h at 4 µM concentration in DMEM complete culture medium before other assays were conducted. The first observation after treatment was that the cell number significantly decreased in HCT-116 cells (Figure 1A vs. 1B), whereas no such change was seen in HT-29 cells (Figure 1C vs. 1D).

This result suggests that S7333 treatment has a specific effect on HCT-116 cells but not on HT-29 cells (Figure 1E). Both cells were then set for tumor spheroid formation after S7333 treatment. Interestingly, the result showed that the number of tumor spheroids was obviously increased after the treatment in HCT-116 but the sphere size was greatly decreased under the microscope, compared to the untreated controls (Figures 1F vs. 1G). The quantitative determination of the total spheroid number (Figure 1H) and size (in terms of average cells/spheroid, Figure 1I) was statistical significance (*p* < 0.001 and *p* < 0.05, respectively). In contrast, there were no such changes observed in HT-29 K-Ras^WT^ cells after S7333 treatment both in spheroid number and size (Figures 1J vs. 1K, and Figures 1L,M). These data collectively suggest that S7333 can effectively inhibit HCT-116 cells that leads to the decrease of their cell proliferation but increase the spheroid formation, an important indicator of cancer stemness.

### Inhibition of K-Ras^G13D^ mutation increases cancer stemness in HCT-116 cells

**FIGURE 2:**
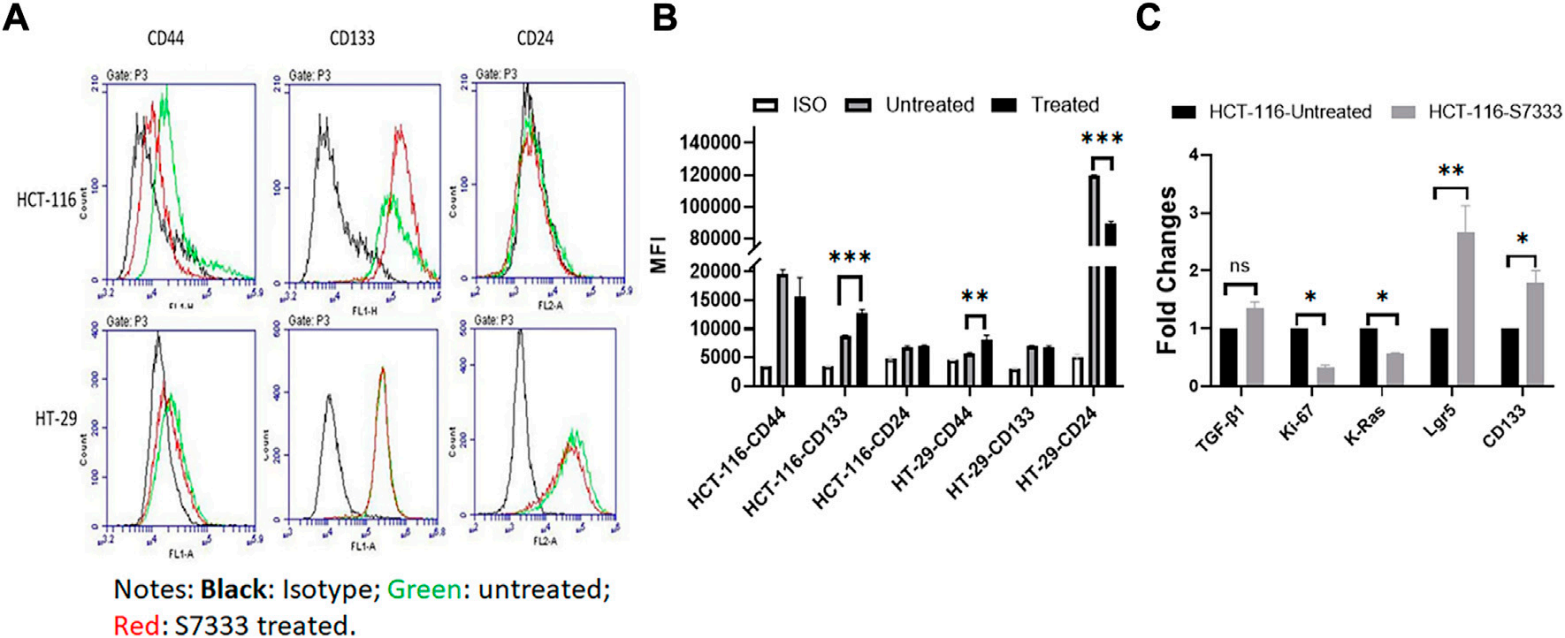
Expressions of stemness markers in HCT-116 and HT-29 after S7333 treatment. The results of flow cytometry analysis show the stemness surface markers CD133, CD44, and CD24 in HCT-116 and HT-29 cells after S7333 treatment **(A)**. For HCT-116 cells, CD133 expression increased while CD44 expression decreased and CD24 remained almost the same. For HT-29 cells, CD44 increased while CD24 decreased and CD133 remained the same **(A)**. **(B)** shows the summary of the flow cytometry results with 3 repeated experiments. **(C)**, Real-time q-PCR results, show that the stem genes CD133 and Lgr5 significantly increase while the self-renewal gene TGF-β1 slightly increases after S7333 treatment. The ability of cell proliferation decreases as indicated by low Ki67 gene expression and K-Ras gene has been suppressed by S7333 treatment. *: *p* < 0.05; **: *p* < 0.01; ***: *p* < 0.001.

To further investigate the increased stemness in HCT-116 cells after S7333 treatment, we examined signatural stem markers of colon CSCs, including the cell surface markers like CD133, CD44, and CD24 and gene markers like Lgr5, CD133, and TGF-β1 before and after S7333 treatment. Ki-67 gene expression was also measured to confirm cell proliferation and K-Ras gene was included to verify the effect of S7333 treatment. The results showed that after the treatment in mutant HCT-116 cells, cell surface CD133 expressions significantly increased in terms of positive cell percentage (Table 1) and mean fluorescent intensity (MFI, Figures 2A,B).

**TABLE 1:** Comparison of the positive rate of cancer stem surface makers between HCT-116 and HT-29 cells before and after S7333 treatment.

	CD44^+^ cells (mean ± SD %)		CD133^+^ cells (mean ± SD %)		CD24^+^ cells (mean ± SD %)	
	HCT-116	HT-29	HCT-116	HT-29	HCT-116	HT-29
Un-treated	38.8 ± 1.4	0.4 ± 0.1	46.4 ± 1.4	10.9 ± 1.1	3.8 ± 0.1	89.2 ± 0.7
S7333 treated	18.4 ± 0.9	0.6 ± 0.1	59.2 ± 1.2	9.8 ± 1.7	3.9 ± 0.1	84.3 ± 2.2

The surface level of CD24 expression also increased slightly but CD44 expression decreased (Figures 2A,B). The CD133 positive cells increased from 46.4% to 59.2%, and CD44 positive cells decreased from 38.8% to 18.4% (Table 1). As a control, HT-29 cells were also assayed for stem markers. As shown in Figure 2A and Table 1, the expressions of these surface markers in K-Ras^WT^ cells were different from HCT-116 cells where significant increase of CD44 and significant decrease of CD24 between the treated and untreated cells were observed (Figure 2B).

Real-time qPCR results further proved that the stem genes of CD133 and Lgr5 expressions significantly increased in treated HCT-116 cells while Ki-67 was decreased after S7333 treatment (Figure 2C). The self-renew gene TGF-β1 expression was also higher after S7333 treatment in HCT-116 cells though did not reach the statistical significance (Figure 2C). As expected, the K-Ras gene expression was significantly down-regulated after S7333 treatment, indicating the inhibitor specifically acted on K-Ras gene of HCT-116 but not HT-29 cells (Figure 2C). Together, all above data suggest that the major cancer stem markers increase but the cell proliferation decreases when K-Ras^G13D^ mutation is suppressed by S7333 treatment.

### Inhibition of RAS/ERK pathway suppresses the spheroid formation and stemness profile in HCT-116 rather than HT-29 cells

**FIGURE 3:**
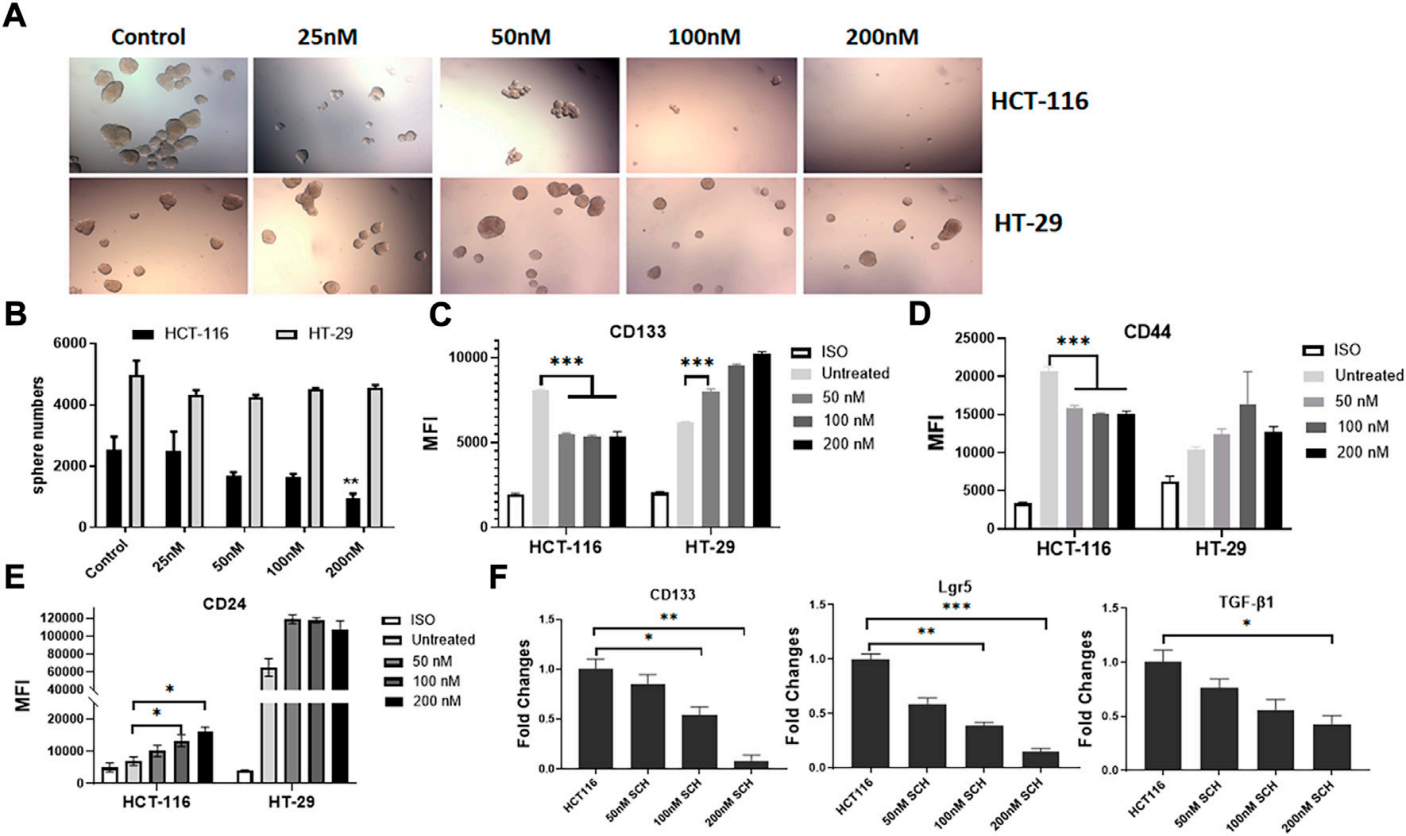
Inhibition of ERK by SCH772984 suppresses the spheroid formation and stemness in HCT-116 but not in HT-29 cells. The photos show the morphology and numbers of tumor spheroids from HCT-116 and HT-29 cells after SCH772984 (SCH) treatment at different doses **(A)**. A dose-dependent inhibition of sphere formation and growth in HCT-116 cells can be seen, comparing to that in HT-29 cells. **(B)**, the number of spheroids was significantly decreased in HCT-116 cells at the 200 nM dose, but not in HT-29 cells. Flow cytometry analysis showed surface marker expressions of CD133 **(C)** and CD44 **(D)** decreased in dose-dependent manners at the doses of 50, 100, and 200 nM in HCT-116, though there was a slight increase for CD24 **(E)**. Inversely, the expressions of these markers increased (C, D, E) in HT-29 cells after SCH772984 treatment. **(F)**, the stem gene expressions of CD133, Lgr5, and TGF-β in HCT-116 cells, they all displayed a dose-dependent decrease after SCH772984 treatment. *: *p* < 0.05; **: *p* < 0.01; ***: *p* < 0.001.

To understand the signaling pathway through which the mutated K-Ras^G13D^ affects the stemness in HCT-116 cells, we analyzed the downstream signal pathways of K-Ras in the cell line and used HT-29 cells as the control. Based on the literature search, there are two typical and well-studied pathways under K-Ras regulation; RAS/ERK and PI3K/Akt pathways (Thein et al., 2021). We firstly tested if ERK pathway inhibitor SCH772984 alone could inhibit the spheroid formation of the two cell lines at the doses from 25 to 200 nM. We found a dose dependent inhibition of spheroid formation in HCT-116 cells but not in HT-29 cells after 48 h treatment (Figure 3A). The spheroid number counts reached statistical significance at the dose of 200 nM (Figure 3B). These data suggest that K-Ras^G13D^ mutated cells are more sensitive to SCH772984 treatment than the wildtype HT-29 cells. In another word, the ERK pathway may be more actively involved in spheroid formation in K-Ras^G13D^ mutated colon cancer cells.

Next, we assessed the expression of surface markers in different dose groups of HCT-116 and HT-29 cells by FACS analysis. In HCT-116 cells, the expression of CD133 and CD44 were remarkably decreased after inhibition of ERK activity by SCH772984 treatment in both positive cell rates (decreased from 52.8% to 35.3% for CD133, and from 74.3% to 29.8% for CD44) and MFI (Table 2 and Figures 3C,D). However, the treatment increased CD24 expression in terms of MFI (Figure 3E) and positive cell rates (Table 2). Differently, in HT-29 cells, the treatment increased MFI of CD133 as the dose increases (Figure 3C), while MFI of CD44 (Figure 3D) and CD24 (Figure 3E) slightly increased with the positive cell rates basically remaining unchanged (Table 2). These findings suggest that in colon cancer, except CD24 expression, ERK pathway differently regulates stemness properties between mutant K-Ras^G13D^ and K-Ras ^WT^ cells, which may relate to the mutation.

**TABLE 2:** Comparison of positive cells (%) of cancer stem surface makers between HCT-116 and HT-29 cells after S7333 or SCH or combined treatment.

	CD44^+^ cells (%)		CD133^+^ cells (%)		CD24^+^ cells (%)	
	HCT-116	HT-29	HCT-116	HT-29	HCT-116	HT-29
Untreated cells (Control)	74.3 ± 9.8	22.6 ± 1.9	52.8 ± 4.8	41.5 ± 6.5	4.8 ± 1.3	94.4 ± 8.3
S7333 treated	78.0 ± 7.7	19.0 ± 2.3	59.3 ± 7.5	42.7 ± 5.6	2.4 ± 1.1	96.1 ± 9.5
SCH treated	29.8 ± 5.4	19.7 ± 3.1	35.3 ± 2.9	73.5 ± 7.9	16.8 ± 3.2	99.0 ± 11.9
S7333+SCH	24.8 ± 3.6	20.5 ± 2.3	34.9 ± 3.3	77.9 ± 7.3	18.6 ± 2.9	97.5 ± 10.7

To further confirm the effect of ERK pathway on the stemness in mutant HCT-116 cells, we measured stem gene expressions after SCH772984 treatment. The results demonstrated that the inhibitor could effectively suppress all stem gene expressions including CD133, Lgr5, and TGF-β1 in HCT-116 cancer cells and in dose-dependent manners (Figure 3F). Among them, CD133 and Lgr5 were more significant than TGF-β (Figure 3F). These data collectively suggest that the inhibition of ERK pathway can decrease the cancer stemness profile in HCT-116 cells more sensitively than in HT-29 cells.

### Blocking K-Ras^G13D^ mutation attenuates the suppressive effect of ERK inhibitor SCH772984

**FIGURE 4:**
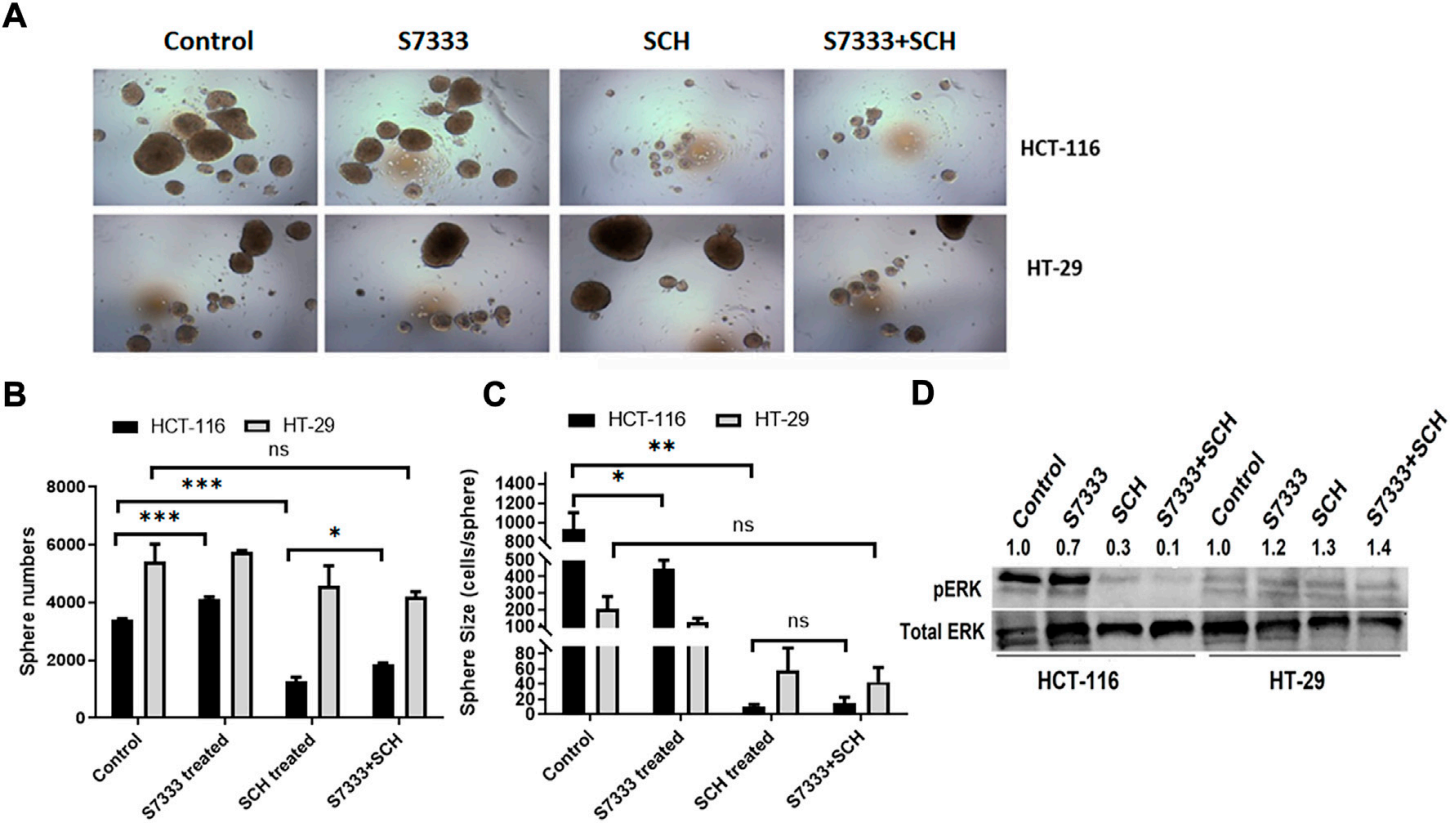
After blocking K-Ras ^G13D^ mutation, the sensitivity to ERK pathway inhibitor SCH772984 reduced in HCT-116 but not HT-29 cells. The images show the sphere formation rates and numbers after the S7333 (4 µM for 72 h) or SCH772984 (200 nM for 48 h) alone and the combination treatment in HCT-116 and HT-29 cells **(A)**. The sphere number counts show the significant increase after S7333 treatment and decrease after SCH772984 treatment, compared with the control **(B)**. However, there is a significant increase of spheroids by SCH772984 treatment after blocking the K-Ras mutation with S7333 **(B)**. The sphere size was obviously decreased after both S7333 and SCH772984 treatment alone **(C)**. However, after blocking K-Ras mutation, SCH772984 treatment slightly increases the size **(C)**. The Western blotting results show the protein levels of phosphorylated ERK (pERK) and total ERK protein (total ERK) in HCT-116 and HT-29 cells after S7333, SCH772984, and combination treatment. The numbers on the protein bands represent densitometry analysis results **(D)**. *: *p* < 0.05; **: *p* < 0.001; ***: *p*< 0.0001; ns: *p* > 0.05*.*

To confirm that the significant inhibition of stemness properties by ERK inhibitor SCH772984 in HCT-116 cells is relevant to K-Ras^G13D^ mutation, we firstly treated HCT-116 with S7333 for 72 h to block the effect of mutation followed by the treatment with the ERK inhibitor SCH772984 at the dose of 200 nM. The results showed that after S7333 treatment the spheroid number increased but their sizes decreased compared to controls (Figure 4A), which are consistent with our above results in Figure 1, suggesting the treatment was effective. However, compared to SCH772984 treatment alone that was shown in the above section to significantly inhibit spheroid formation, did not show such inhibition in both sphere number (in fact significantly increased) and size (slightly increased, Figure 4A). In contrast, no significant changes were seen in spheroid numbers of K-Ras^WT^ cell line HT-29 (Figures 4A,B). For tumor spheroid sizes, in terms of average cell numbers per spheroid, both SCH772984 alone and S7333 + SCH772984 combination treatment decreased (Figure 4C). Notably, same as the spheroid number.

The size did not decrease in the combination treatment group, compared to SCH772984 alone group (Figure 4C). To further prove that S7333 and SCH772984 treatment had effect on the targeted proteins, we conducted Western-blot analysis. As shown in Figure 4D, the expression of phosphorylated ERK (pERK) decreased in HCT-116 cells after S7333 treatment, compared to the control. SCH772984 treatment alone remarkably decreased pERK expression in the cell line harboring K-Kas^G13D^ mutation. The combination treatment with S7333 and SCH772984 further decreased the level of pERK expression. Compared to HCT-116 cells, we observed less inhibitory effects of pERK levels in the cell line with HT-29^WT^ cells (Figure 4D) either with SCH772984 treatment alone or S7333 and SCH772984 combination treatment. These data suggest that the inhibitor treatments can affect both cell lines but more sensitively affect the K-Ras^G13D^ mutated HCT-116 cells.

**FIGURE 5:**
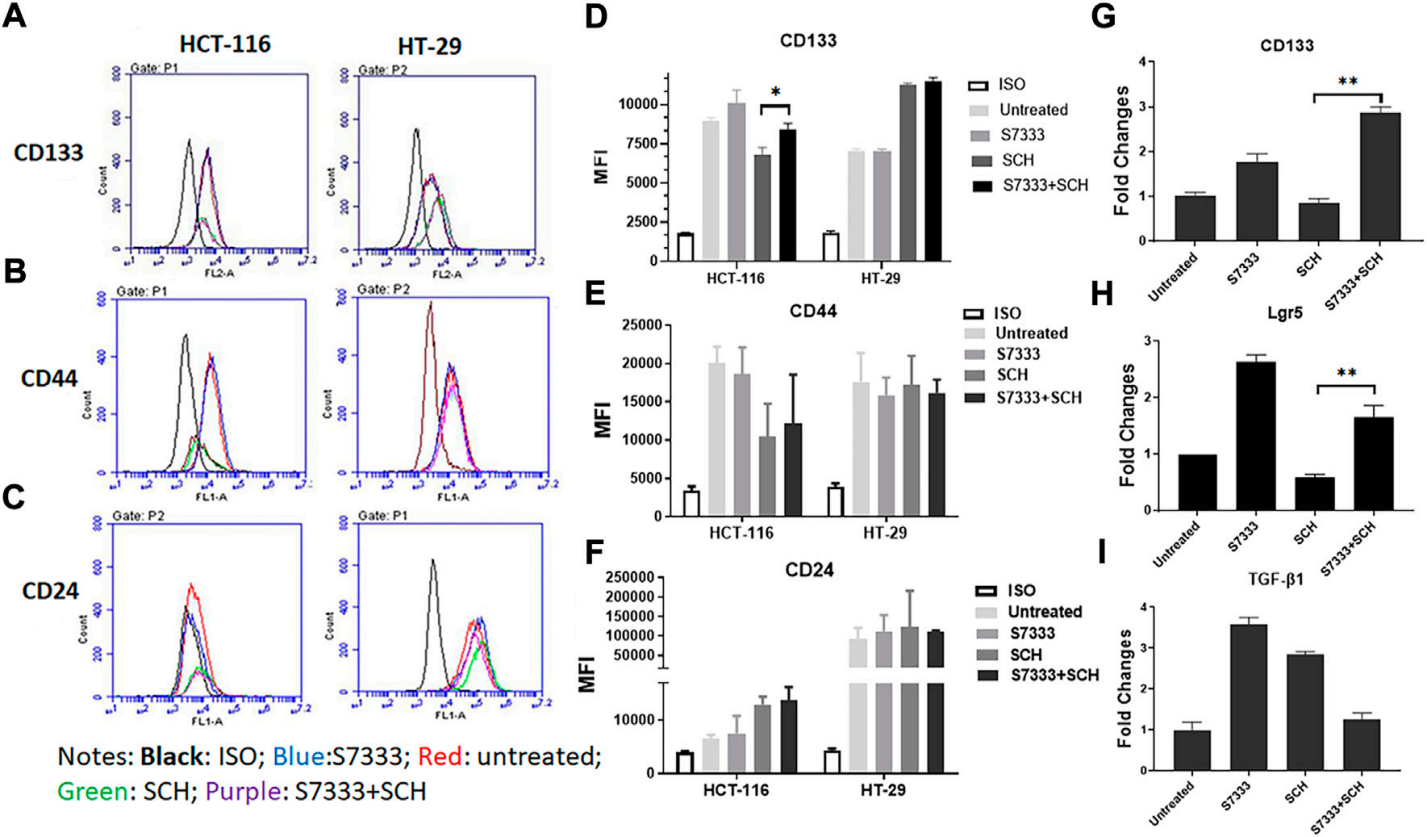
Blocking K-Ras mutation reduced the suppressive effect of SCH772984 treatment on cancer stemness markers in HCT-116 cells. The flow cytometry analysis of the expressions of surface stem markers CD133 **(A)**, CD44 **(B)**, and CD24 **(C)** in HCT-116 and HT-29 spheroids following the SCH772984 treatment after the K-Ras ^G13D^ mutation has been blocked. CD133 and CD44 expressions are increased after the combination treatment whereas the expression of CD133, CD44, and CD24 did not increase after the same treatment in HT-29 spheroids **(D–F)**. Real-time qPCR results show the stem gene CD133 **(G)**, Lgr5 **(H)**, and TGF-β1 **(I)** expressions for HCT-116 cells. *: *p* < 0.05; **:*p* < 0.01.

To further verify ERK pathway involvement in the regulation of cancer stemness, we analysed the stem surface markers of CD133 (Figures 5A,D), CD44 (Figures 5B,E), and CD24 (Figures 5C,F). The results showed that after the treatment of S7333, CD133 level increased whereas SCH772984 treatment decreased the level. After blocking the K-Ras^G13D^ mutation, the SCH772984 treatment significantly increased CD133 expression (Figure 5D). For CD44, S7333, and SCH772984 treatment alone decreased the expression but SCH772984 treatment after S7333 blocking the mutation slightly increased the expression levels (Figure 5E). For CD24, either treatment alone with S7333 and SCH772984 or the combination treatment increased the expression levels (Figure 5F). In contrast, in HT-29 cells, the CD133, CD44, and CD24 expressions were different from HCT-116 cells in either treatment alone with S7333 and SCH772984 or combination treatment with both (Figures 5A–F). These results suggest even after blocking K-Ras^G13D^ mutation, there was a difference in signalling pathways in the two cell lines.

To further verify the stemness in HCT-116 cells, we measured the stem gene expression. The results showed that after blocking K-Ras^G13D^ mutation in HCT-116 cells, SCH772984 treatment did not decrease but increase stem gene profiles including CD133 (Figure 5G) and Lgr5 (Figure 5H) expressions. However, TGF-β1 (Figure 5I) expression decreased after the combination treatment. Consistent with the results in Figure 2, after S7333 treatment, CD133, Lgr5, and TGF-β1 gene expression all increased, compared to the control (all *p* < 0.05, Figures 5G–I). Taken together, all above data indicate that after blocking K-Ras^G13D^ mutation, ERK inhibitor has attenuated its suppressive effect on HCT-116 cells but not much in HT-29 cells, that implies that K-Ras^G13D^ mutation works *via* the ERK pathway to promote major cancer stemness in HCT-116 cells.

### PI3K/Akt/mTOR pathway inhibition equally suppresses spheroid formation in both cell lines

**FIGURE 6:**
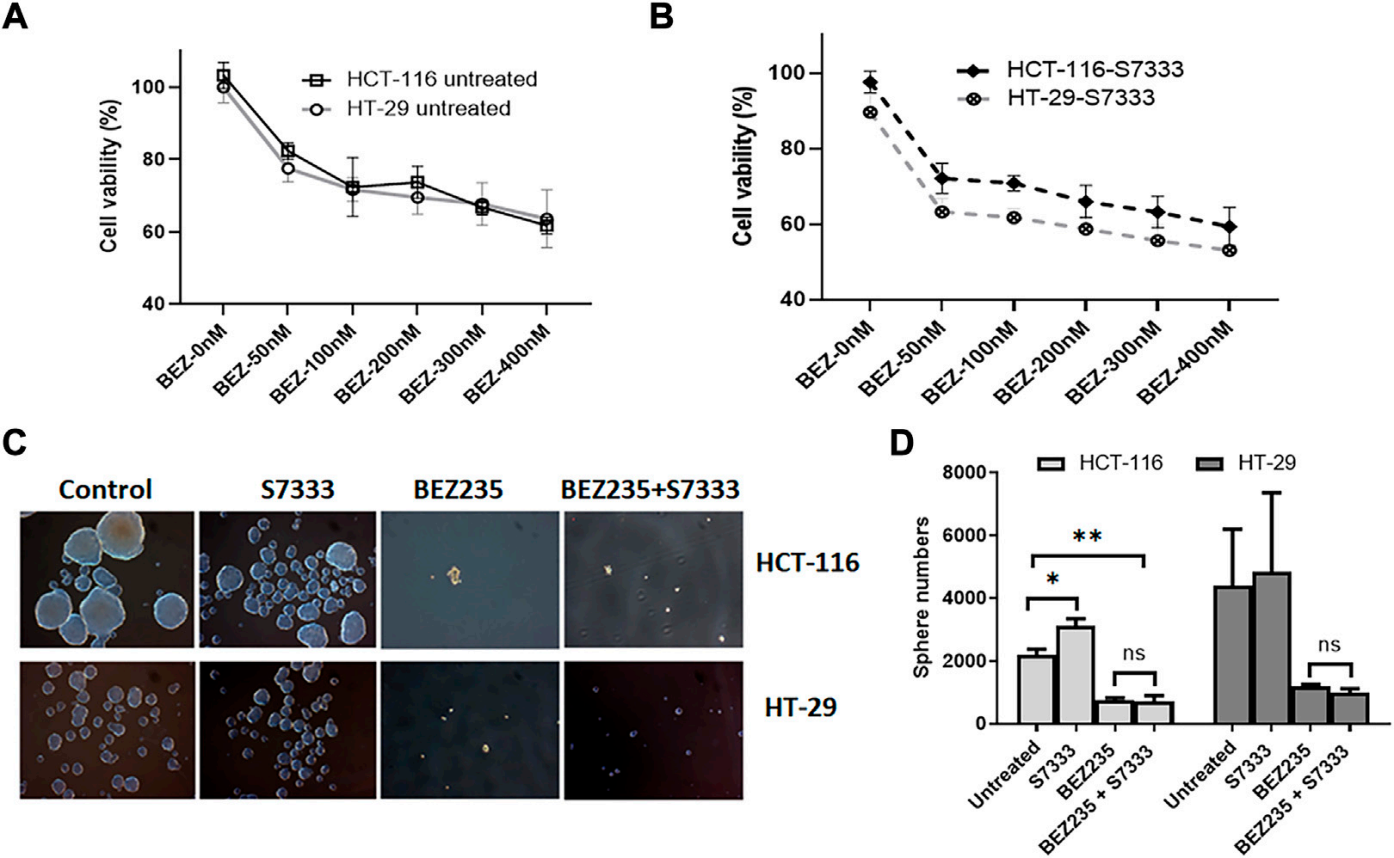
BEZ235 can equally inhibit the cancer stemness in HCT-116 and HT-29 cells. Cell viability assay of MTT shows that both HCT-116 and HT-29 cells exhibit a similar dose-dependent decrease as BEZ235 dose increases **(A)**. After S7333 treatment, they still exhibit the dose-dependent decrease of cell viability though HT-29 cells are slightly more sensitive than HCT-116 **(B)**, but there are no statistical differences between these two cell lines at any doses. The sphere numbers were remarkably decreased after BEZ235 treatment with or without S7333 blocking the K-Ras^G13D^ mutation in both HCT-116 and HT-29 cells **(C,D)** . *: p < 0.05; **: p< 0.01*.*

Another axis under K-Ras regulation is PI3K/Akt/mTOR pathway. To investigate if this pathway was also involved in K-Ras^G13D^ regulation, we used a dual inhibitor of this pathway BEZ235 that targets both PI3K and mTOR (Chen et al., 2014). We treated HCT-116 and HT-29 cells with BEZ235 at different doses and measured cell viability using the MTT assay. The results showed that both cells exhibited a dose-dependent decrease of cell viability and there was no difference between the two cell lines at the doses ranged from 50 to 400 nM (Figure 6A). We also measured their cell viability after S7333 treatment, the results showed that both cells exhibited a similar dose-dependent decrease of cell viability with HT-29 cells being slightly more sensitive than HCT-116 to the treatment (Figure 6B). However, there was no significant difference between the two cell lines. In addition, BEZ235 treatment significantly suppressed the sphere formation in both cell lines no matter they were treated with S7333 or not (Figures 6C,D). These data suggest that BEZ235 is effective at suppressing PI3K/Akt pathway in colon cancer cells with or without K-Ras^G13D^ mutation. This implicates that, unlike the ERK pathway, PI3K/Akt pathway is independent of the regulation of K-Ras^G13D^ mutation.

**FIGURE 7:**
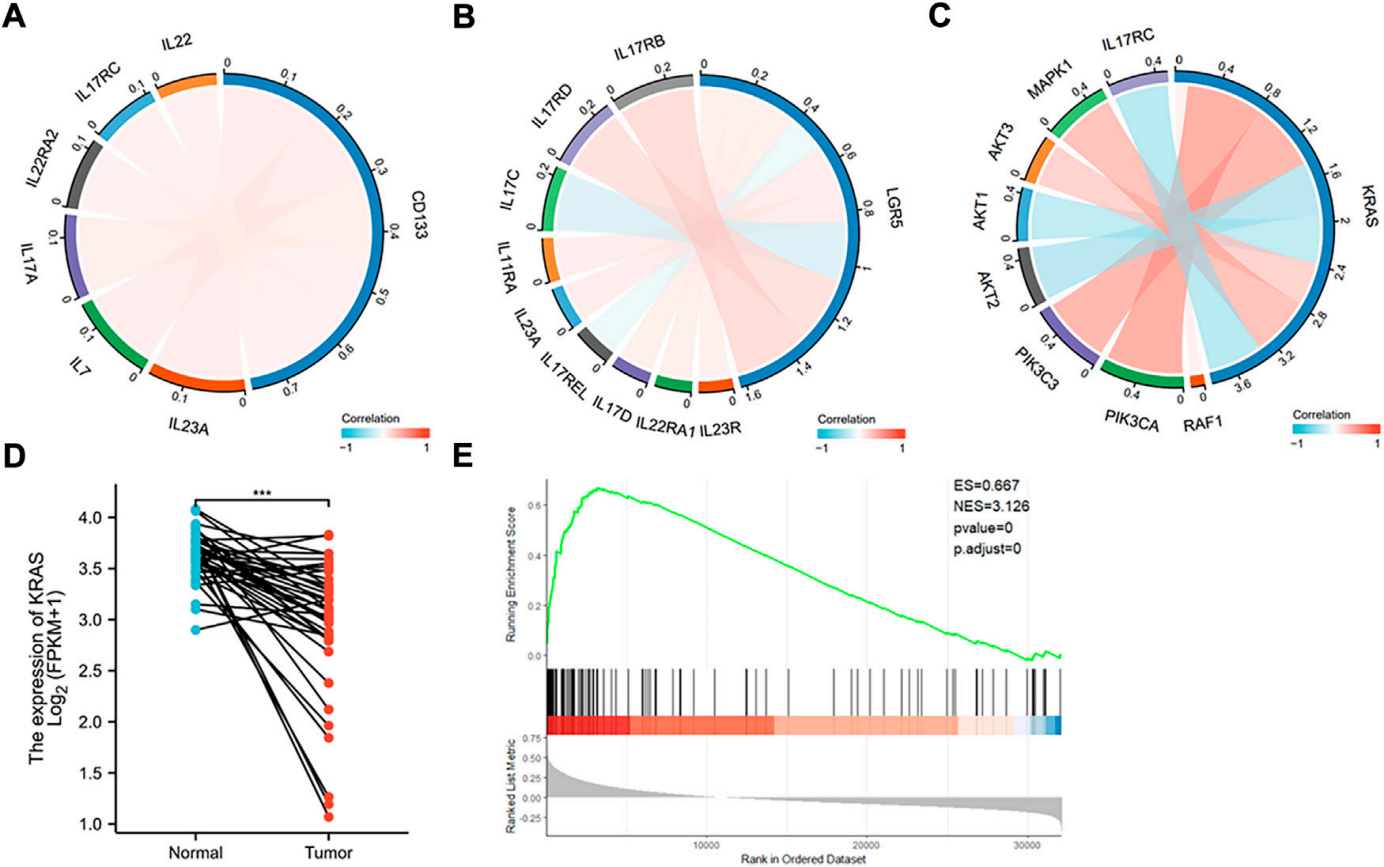
Database analysis of stem and K-Ras gene expressions in colon cancer. **(A)** Chordal graph of the correlation between the expression of CD133 and inflammatory genes. The expression of CD133 was found to positively correlate with IL-7, IL-17A, IL-17RC, IL-22RA2, IL-22, and IL-23A. **(B)** Chordal graph of the correlation between the expression of Lgar5 and inflammatory genes. The expression of LGR5 was found to positively correlate with IL-11RA, IL-17RB, IL-17RD, IL-17D, IL-22RA1, IL-23R, and IL-23A, and negatively correlate with IL-17C, and IL-17REL. **(C)** Chordal graph of the correlation between the expression of K-Ras and inflammatory genes. The expression of K-Ras was found to positively correlate with RAF1, PIK3CA, PIK3C3, AKT3, and MAPK1, and negatively correlate with AKT1, AKT2, and IL-17RC. **(D)** K-Ras gene expression based on the TCGA database analysis in normal and colon cancer tissues (***: *p* < 0.001). **(E)** GSEA analysis indicated significant correlations between K-Ras mRNA expression and stemness-related gene signatures. GSEA from the publicly available GEO database (Colon cancer) has shown positive correlation (*p* < 0.05) between K-Ras expression and stemness-related gene signatures.

To further explore if the increased stemness in colon cancer will relate to tumor recurrence and metastasis, we employed the public database to analyze the correlation between CD133 and Lgr5 and other relevant gene expressions. Interestingly, we found that both stem genes were associated with gene expressions of some pro-inflammatory cytokines like IL-17, IL-22, and IL-23 (Figures 7A,B). These cytokines have been confirmed for the roles in tumor development “inflammation and tumor transformation”, and they mostly play a promotive role in tumor initiation and progression (Grivennikov et al., 2012). This analysis indicates that cancer stemness may involve in the regulation of inflammation potential that helps CSCs to start tumor initiation and “inflammation and tumor transformation”.

We then analyzed the expression of K-Ras with possible signaling pathways, it was found that it positively correlated with RAF1, PIK3CA, PIK3C3, AKT3, and MAPK1, and negatively correlated with AKT1, AKT2, and IL-17RC (Figure 7C). These pathways are mostly related to cell survival and inflammation (Zhao et al., 2021). We also analyzed K-Ras mRNA expression in normal and cancer samples of colon cancer patients (Figure 7D) and found that K-Ras mRNA expression in cancer tissues was significantly lower than in normal tissues, suggesting that K-Ras itself (without mutation) may be not a promotive factor for inflammation. However, when it mutates (at least G13 mutation) it will regulate cancer stemness. If the mutation has being suppressed, it will increase the stemness like CD133 and Lgr5 levels and subsequently promote inflammation potential. To further prove this, we analyzed K-Ras and its relationship with cancer stemness by conducting the gene set enrichment analysis (GSEA) from the publicly available GEO database (for colon cancer). The result showed a positive correlation (*p* < 0.05) between K-Ras expression and stemness-related gene signatures (Figure 7E). This analysis supports a possible link between K-Ras expression and cancer stemness.

## Discussion

K-Ras, a GTPase, has a high frequency (∼30%) of mutants occurred in all human cancers, and the association with the cancer aggressiveness and resistance to existing therapies. In colorectal cancer, around 40% of cancer cases harbor a K-Ras mutation that dictates the resistance to therapies and poor prognosis (Morkel et al., 2015; Blaj et al., 2017). Consequently, inhibitors targeting K-Ras mutations, especially the K-Ras G12C mutation, have been developed and proceeded to phase I/II clinical trials with promising results (Seton-Rogers, 2020). However, available information indicates that each K-Ras mutation (including codons 12 and 13) has a distinct gene/protein profile and is very different in signal pathway activations (Hammond et al., 2015), suggesting a deep understanding of the molecular mechanism of each mutation is essential for guiding more precise and effective treatments of K-Ras mutated colon cancer patients in clinic. To achieve this, in this study we attempted to understand the relation between K-Ras^G13D^ mutation and cancer stemness, as CSCs are majorly responsible for tumor initiation, development, drug-resistance, and metastasis. Our data indicate that suppressing K-Ras^G13D^ mutation in colon cancer cells with a pre-clinical G12C inhibitor S7333 can reduce the cell proliferation but promote their stemness including spheroid formation, stem marker CD133, K-Ras, and stem gene Lgr5 expressions. In contrast, no such increases are seen in K-Ras wildtype cells after the same treatment, confirming that S7333 treatment in HCT-116 is not an off-target effect on K-Ras gene and that K-Ras^G13D^ mutation plays a role in increasing cancer cell proliferation and suppressing cancer stemness in colon cancer. These findings thus prove that K-Ras^G13D^ mutation can regulate cancer stemness and therefore contribute to tumorigenesis of colon cancer. Our database analysis reveals that compared to normal tissues, colon cancer samples express less K-Ras mRNA (Figure 7D), suggesting that mutation could be the mechanism that the K-Ras gene promotes colon cancer growth.

According to the manufacturer, S7333 is a K-Ras G12C selective inhibitor. However, in our study, we treated the cells with S7333 over 72 h at a dose of 4 µM to ensure G13D mutation has been blocked. Our data have confirmed this though more detailed data on G13D mutation detection or employing HCT-116 wildtype control cells will provide further confirmations.

**FIGURE 8:**
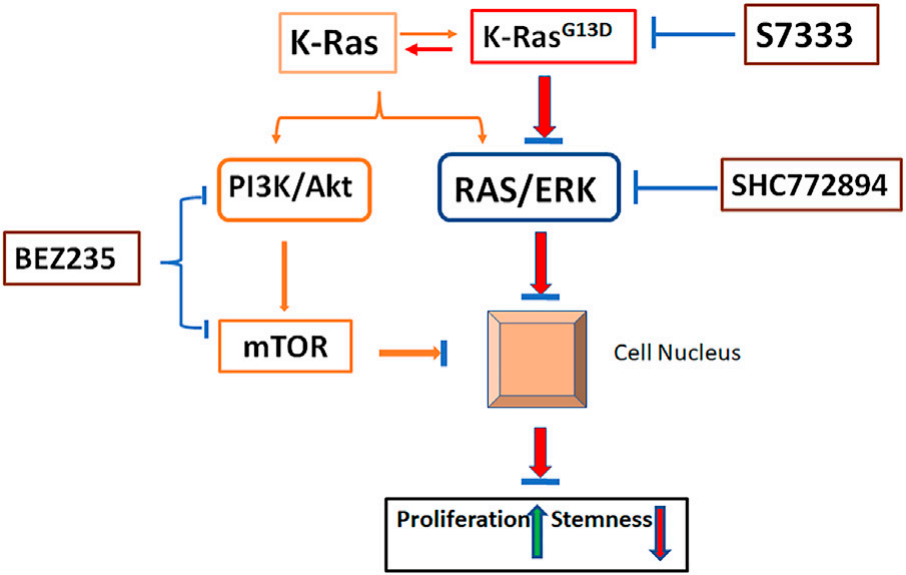
Schematic illustration of pathways involved in K-Ras^G13D^ mutation. In the status of wild type, K-Ras activates both PI3K/Akt and RAS/ERK pathways and promote cancer cell growth while suppress stemness (orange lines). Upon mutation at K-Ras^G13D^, it mainly acts on RAS/ERK pathway to promote cell proliferation and reduce stemness by regulating gene expressions in the nucleus (Red lines). Inhibiting the mutation by S7333 will suppress RAS/ERK pathway (blue lines). Under the activation of K-Ras^G13D^ mutation, the treatment of SCH772894 can more effectively suppress the RAS/ERK pathway thus cell proliferation and stemness in mutated but not wild type cells (blue lines). Differently, BEZ235 treatment can inhibit cell growth and stemness in both mutated and un-mutated cells *via* PI3K/Akt and bypassing RAS/ERK pathway. After blocking K-Ras^G13D^ mutation, the activating signal of RAS/ERK is reduced, the sensitivity to SCH772894 treatment in mutated cells decreases.

Specific point mutation usually links to certain signaling pathway alterations. Of the two major pathways positively related to K-Ras, we have shown that K-Ras^G13D^ mutation mainly associates with RAS/ERK rather than the PI3K/Akt pathway (Figure 8). As more and more inhibitors for K-Ras mutation proceed to pre-clinical studies or clinical trials, the importance of this study becomes more obvious and is necessary to consider in clinical practice. That is, the K-Ras targeted therapy (with inhibitors), though can inhibit the cancer cell growth, may promote cancer stemness thus maintain CSCs and inflammation potential that will lead to tumor recurrence and metastasis. In this case, according to our results, for K-Ras^G13D^ mutant colon cancer, PI3K/Akt/mTOR or RAS/ERK pathway inhibitor (BEZ235 or SCH772984) alone would be a better treatment option than S7333 alone. When combinational therapy is necessary, the combination of S7333 with BEZ235 may be a better choice than with SCH772984. However, these concepts need to be tested in clinal trails.

Unlike G12C, that is, the most frequently observed and studied K-Ras mutation in colon cancer, G13D mutation is much less studied and no specific inhibitor has been reported. We therefore used the S7333 inhibitor to investigate its effect on cancer stemness. Our data suggest that the effect on increasing cell proliferation and reducing cancer stemness was mainly *via* the RAS/ERK pathway. Although the role of RAS/ERK pathway in cell survival and proliferation has been well-described (Lavoie et al., 2020; Asl et al., 2021), there are not many studies describing its link with cancer stemness. Wang et al. (2010) reported that the upstream molecules in Akt and MAPK pathways were preferentially expressed in colon cancer CD133 (+) cells and the kinase activities of Akt and (ERK)1/2 were also significantly upregulated in CD133 (+) cells. The treatment of synovial sarcoma Fuji cells with a specific MEK/ERK inhibitor, U0126, markedly decreased CD133 expression, but there was no significant effect in colon cancer Caco-2 cells, suggesting cell type-specific regulation of CD133 expression. Instead, the side population (another hallmark of CSCs) was dramatically diminished in Caco-2 cells by U0126 (Tabu et al., 2010), which is similar to our results on the spheroid formation (Figure 3). In addition, Ras-mediated oncogenic transformation in normal human astrocytes conferred the stem-like capability to form neurosphere-like colonies.

Neurosphere-like colonies with the increase of CD133 mRNA expression (Tabu et al., 2010). Therefore, the RAS/ERK pathway at least in part contributes to the maintenance and acquisition of stem-like hallmarks, although the extent of its contribution is varied in a cell type-specific manner. A study examined the inhibitory effect of tubulin inhibitor STK899704 on colon cancer cell migration and CSC stemness and showed that STK899704 downregulated the mRNA expression levels of the cell migration mediator focal adhesion kinase (FAK) and suppressed MAPK and ERK, which are downstream signaling molecules of FAK. Additionally, STK899704 inhibited stem gene expression and sphere formation in colon cancer stem cells (Jang et al., 2018), suggesting that ERK can involve in regulating stemness in colon cancer, which is consistent with and confirmed by our current study.

Above information suggests that RAS/ERK pathway can maintain or regulate cancer stemness in colon cancer, which is particularly true in K-Ras^G13D^ mutated colon cancer where the mutation more specifically activated on ERK. Therefore, we can see more suppressive effects on stemness (Figure 3) and pERK protein levels (Figure 4D) in HCT-116 than HT-29 cells. When the mutation was blocked by S7333, the treatment of ERK inhibitor was less effective in HCT-116 cells on their stemness (Figures 4, 5). Instead, PI3K/Akt inhibitor BEZ235 treatment was equally effective on both mutated and wildtype cell lines, reflecting the mutation mainly acts on RAS/ERK pathway. Not only in cell lines, K-Ras-ERK axis engagement in cancer stemness or stem cells is also reported in animal models. A study showed the transient expression of reprogramming factors in K-Ras mutant mice was sufficient to induce the robust and persistent activation of ERK signaling in acinar cells and rapid formation of pancreatic ductal adenocarcinoma. In contrast, the forced expression of acinar cell-related transcription factors inhibited the pancreatitis-induced activation of ERK signaling and development of precancerous lesions in K-Ras-mutated acinar cells (Shibata et al., 2018). Not only in epithelial cancers, in human hematopoietic malignancies, RAS mutations are frequently observed and related to cancer stemness. For example, when cultured on bone marrow stroma, K-Ras (G12 V)-transduced cord blood CD34 (+) stem/progenitor cells displayed a strong proliferative advantage over control cells, which coincided with increased early cobblestone (CAFC) formation and induction of myelomonocytic differentiation. Both the ERK and p38 MAPK pathways, but not JNK, were activated by K-Ras (G12 V) and the proliferation and CAFC formation were mediated *via* ERK, while differentiation was predominantly mediated *via* p38 (Fatrai et al., 2011). Above data reveal that K-Ras and its mutations are widely involved in RAS/ERK pathway and cancer stemness.

As to K-Ras^G13D^ mutation, a study reported that the tumor suppressor NF1 was co-mutated in K-Ras G13-mutated cells. NF1 GTPase-activating protein was inactive against K-Ras G12 and Q61-mutated K-Ras but stimulated GTP hydrolysis when bound to K-Ras^G13D^. K-Ras^G13D^ mutant cells also respond to EGFR inhibitors in a neurofibromin-dependent manner. Crystallographic analysis of wild-type and G13D K-Ras complexed with neurofibromin provides the structural basis for neurofibromin-mediated GTP hydrolysis (Rabara et al., 2019). However, this study did not report on whether this mutation is related to cancer stemness, our data thus provide the first evidence of the link. Though the suppression of K-Ras mutation can promote cancer stemness, the direct transfection of K-Ras mutation itself did not promote cancer stemness in colon cancer development. A study showed that K-Ras mutation (G12D) could promote serrated and hyperplastic morphologic features in colon epithelium, but it was not able to initiate adenoma development, perhaps in part because activated K-Ras signaling did not increase the number of presumptive stem cells in affected crypts (Feng et al., 2011). This study suggests that K-Ras mutation may initiate epithelial cell transformation through altering signaling pathways or working with other mutations in the cells. According to our results, we believe that there is another reason for this, that is, the transfected K-Ras mutations mainly promote cell proliferation and suppress major cancer stemness. Nevertheless, in our study, we found that the suppression of G13D mutation promoted major stem markers like CD133, Lgr5, and TGF-β but not others like CD44 and CD24, which were reported as common and important biomarkers for colon cancer by us (Chen et al., 2015) and others (Lee et al., 2015; Ribeiro et al., 2016). This suggests that regulation of colon cancer stemness is more complex and RAS/ERK pathway only involves major stemness regulation. Another possible reason for CD24 did not show meaningful results is that the antibody used in the current study did not stain higher cell populations in HCT-116 cells (Tables 1, 2), whereas our previous study showed a positive population about 17% (Chen et al., 2015). The reason of inconsistency is not clear but could relate to the change of antibody batches. Additionally, CD44 expression increased in HT-29 cells after S7333 treatment in comparison with the decrease in HCT-116, which could be an interesting point to follow.

Despite of CD24 and CD44, CD133 is the molecule playing an initial role in colon cancer cluster formation (Grunt et al., 2015), and is also an essential and more common stem marker of many organs and cancers (Grosse‐Gehling et al., 2013; Jang et al., 2017). In this study, we showed that both mRNA and protein levels of CD133 increased after suppression of K-Ras^G13D^ mutation (Figure 2), suggesting this is an important CSC marker related to spheroid formation. Lgr5 is another stem gene for intestine epithelial cells and is often highly expressed in colon cancer patients. Lgr5 expression in the epithelium and stroma was also closely associated with tumor stage, by integrating functional experiments with Lgr5-sorted cell RNA sequencing data from adenoma and normal organoids, the correlations between Lgr5 and colorectal cancer-specific genes, indicating Lgr5 is an essential marker for studying stem cells in human tissue homeostasis and carcinogenesis (Dame et al., 2018). TGF-β is considered as an EMT and self-renewal marker (Gu et al., 2011). Though it has not reached statistical significance in our study, we showed that this growth factor increased after suppression of *K-Ras* ^*G13D*^ mutation (Figure 2), and it responded to ERK inhibitor and combinational treatments, indicating the self-renewal ability of colon cancer cells was regulated by the mutation. TGF-β pathway is also involved in tumorigenesis of colon cancer, as genetic inactivation of TGF-β type 1 receptor (Tgfbr1/Alk5) enhanced the ability of K-Ras (G12D/+) mutation to drive dedifferentiation and markedly accelerated tumorigenesis, which was associated with a marked activation of MAPK signaling (Cammareri et al., 2017). This suggests that more detailed study is needed for understanding its role in K-Ras^G13D^ settings. Collectively, above data suggest that though some colon cancer stem markers are not modulated by the inhibitor treatments, the major stemness profile aligns well with colon cancer stemness property, therefore supporting the conclusion that suppression of *K-Ras* ^*G13D*^ mutation promotes major cancer stemness in colon cancer.

## Conclusion

In this study, we used an inhibitor to suppress K-Ras ^G13D^ mutation in colon cancer HCT-116 cells and found that the inhibition reduced the cell proliferation in terms of cell numbers and gene expression of Ki-67. However, this also induced an increase of stemness and potency of inflammation in treated cells. In contrast, these changes were not seen in wildtype HT-29 cells, suggesting these alterations only relate to K-Ras^G13D^. Our further investigations indicated that these changes were through ERK but not PI3K/Akt pathway. These data imply that caution needs to be taken when using K-Ras mutation targeted therapy as it may promote cancer stemness and inflammation thus lead to tumor recurrence and metastasis.

## Data Availability

The original contributions presented in the study are included in the article/supplementary material, further inquiries can be directed to the corresponding authors.
