# Supplementary material for: Inhibition of colon cancer K-RasG13D mutation reduces cancer cell proliferation but promotes stemness and inflammation via RAS/ERK pathway
Source: Front Pharmacol. 2022 Oct 28;13:996053. doi: 10.3389/fphar.2022.996053 (PMC9650442; doi:10.3389/fphar.2022.996053)

The original Western blotting membranes for analyzing ERK expression.

1). p-ERK first try:

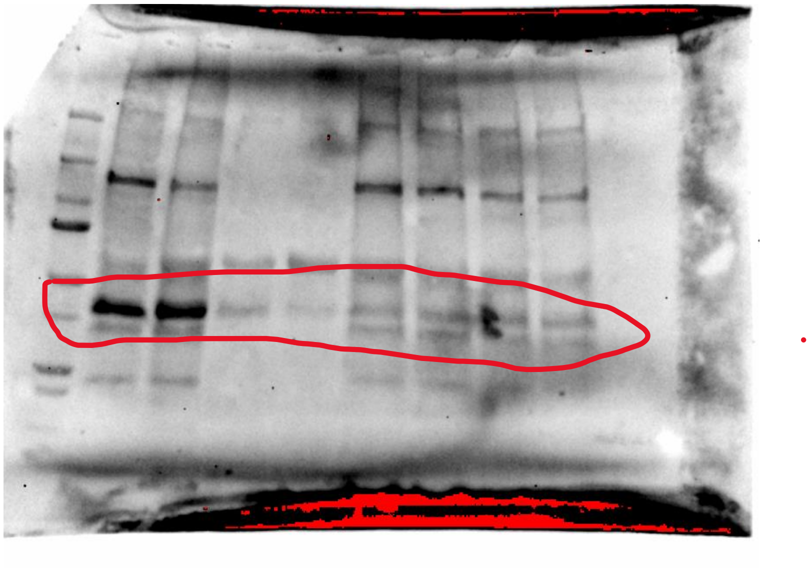

2). p-ERK 2nd try:

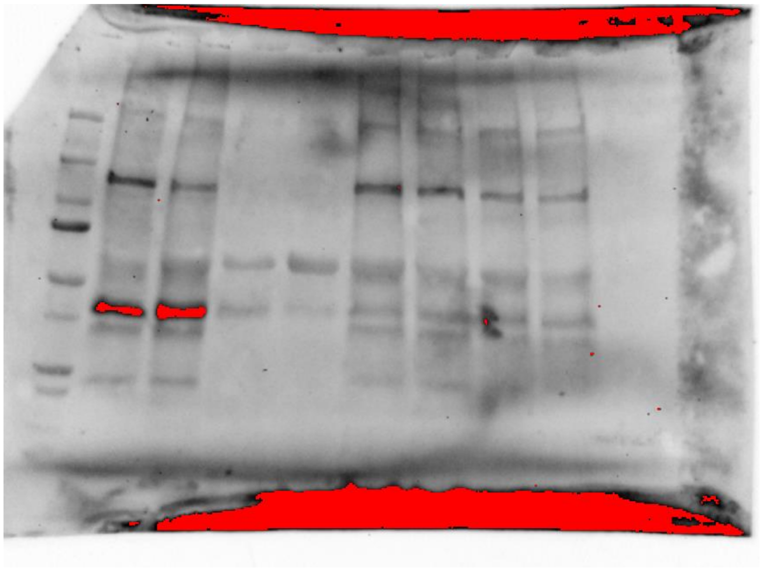

3). Total ERK first try:

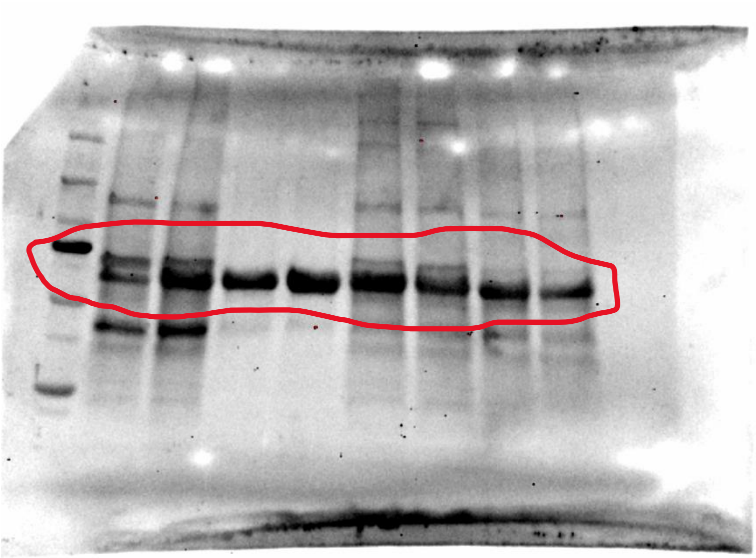

4). Total ERK 2nd try:

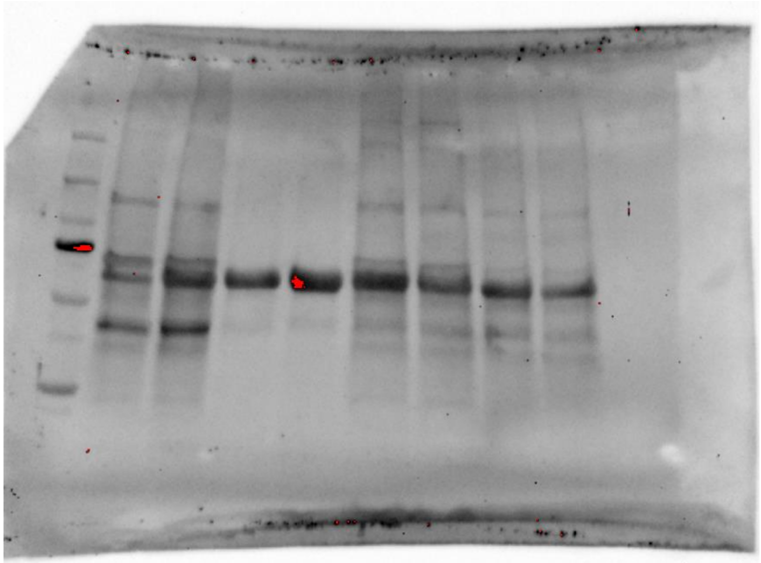

Supplement: Supplementary file 1 [file DataSheet1.PDF]
